# Supplementary material for: Bumble bee microbiota shows temporal succession and increase of lactic acid bacteria when exposed to outdoor environments
Source: Front Cell Infect Microbiol. 2024 Mar 4;14:1342781. doi: 10.3389/fcimb.2024.1342781 (PMC10945022; doi:10.3389/fcimb.2024.1342781)
Supplement: Supplementary file 1 [file DataSheet_1.pdf]

Supplementary Material

Bumble bee microbiota shows temporal succession and increase of lactic acid bacteria when exposed to outdoor environments

Arne Weinhold[1*], Elisabeth Grüner[1], Alexander Keller[1]

[1]Cellular and Organismic Networks, Faculty of Biology, Center for Organismic Adaptation, Ludwig-Maximilians-Universität München, 82152 Planegg-Martinsried, Germany

Supplemental Figures

Supplemental figure S 1 Phylogenetic tree of major ASVs obtained from the bumble bee gut-microbiota (*B. terrestris*).
Supplemental figure S 2 Food plant diversity had no influence on the gut-microbiota diversity of the large earth bumble bee (*B. terrestris*).
Supplemental figure S 3 Comparison of the bumble bee gut-microbiota of foraging worker with adults and larvae sampled from inside the colony.
Supplemental figure S 4 ASV strain turnover within Lactobacillaceae over time.

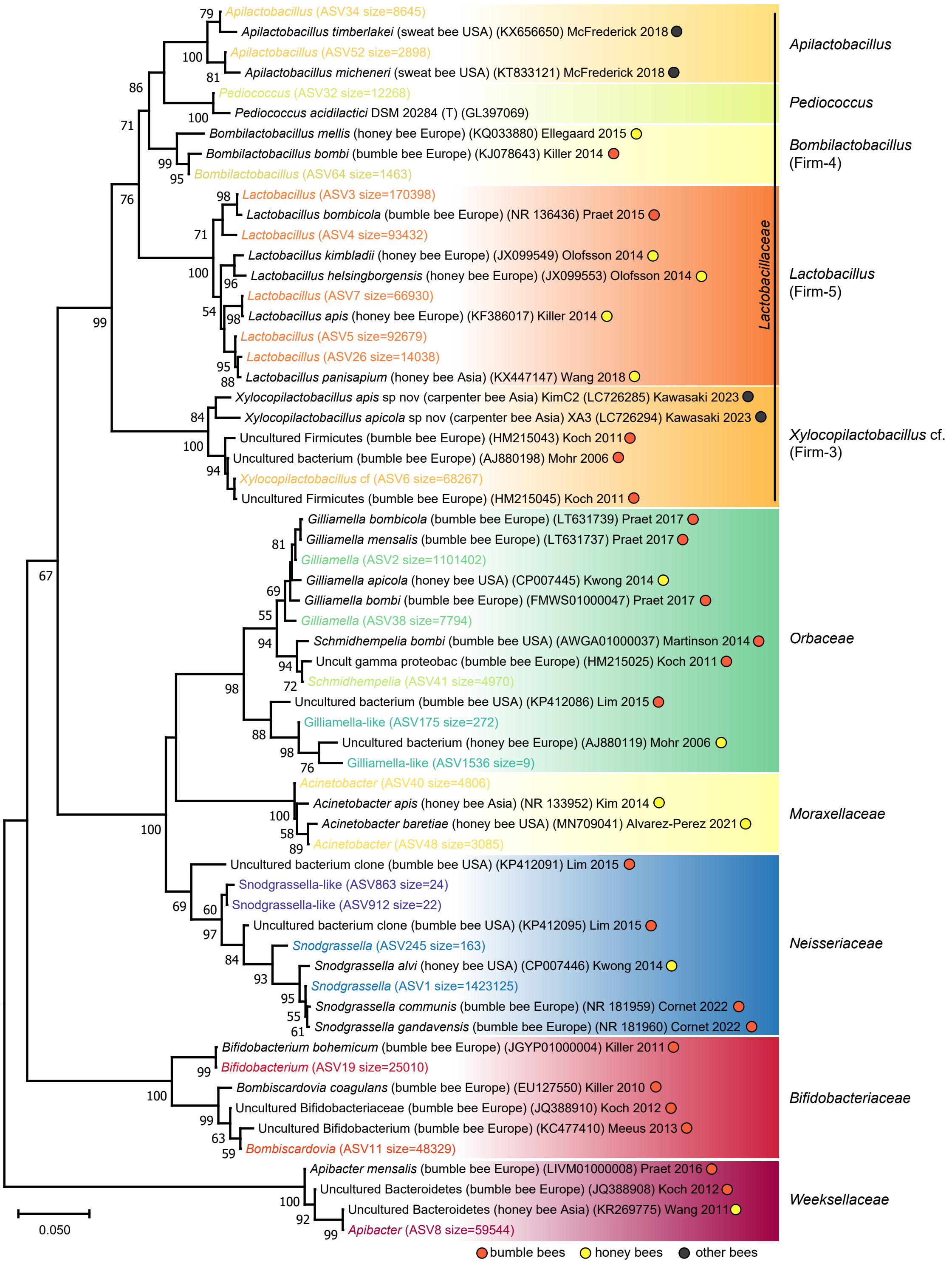

Supplemental figure S 1 Phylogenetic tree of major ASVs obtained from the bumble bee gut-microbiota (*B. terrestris*).
Bumble bee ASVs (in color) were aligned to the closest matching sequences obtained from the NCBI Nucleotide Collection database. Isolation source, geographic origin and references are indicated for each sequence. Neighbor-Joining tree was constructed with MEGA11 and bootstrapping values >50 with 1000 repetitions are shown next to the branches.

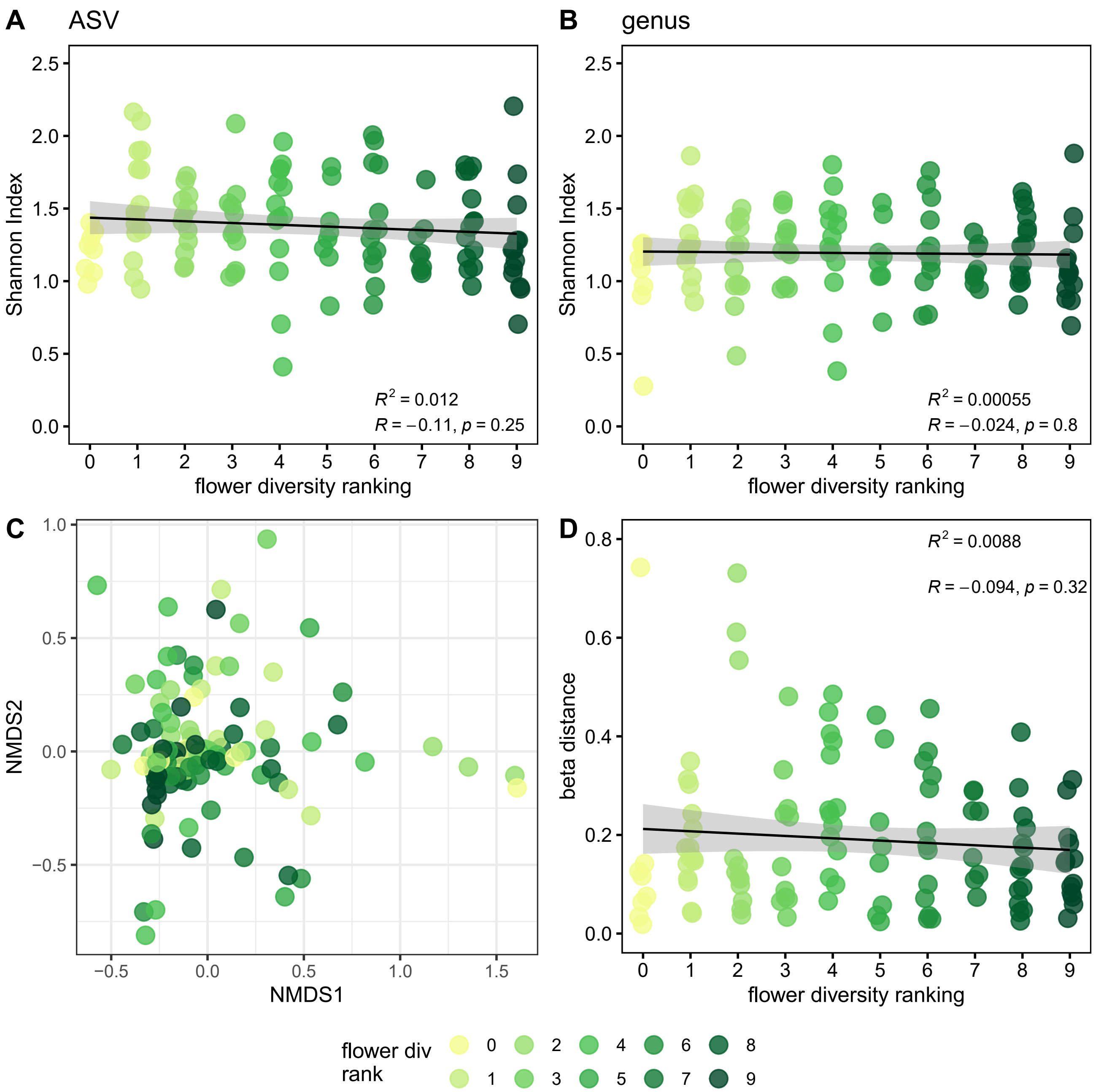

Supplemental figure S 2 Flower diversity had no influence on the gut-microbiota of the large earth bumble bee (*B. terrestris*).

Shannon diversity of the bumble bee gut-microbiota on ASV (A) and genus level (B) shown by flower diversity ranking. Bray-Curtis distance of the bumble bee microbiota shown as NMDS plot (C) and beta distance (D), colored by the flower diversity ranking. Bumble bee worker were sampled from colonies reared in ten individual outdoor flight cages ranked from low to high flower diversity (0 to 9) of provided food plants.

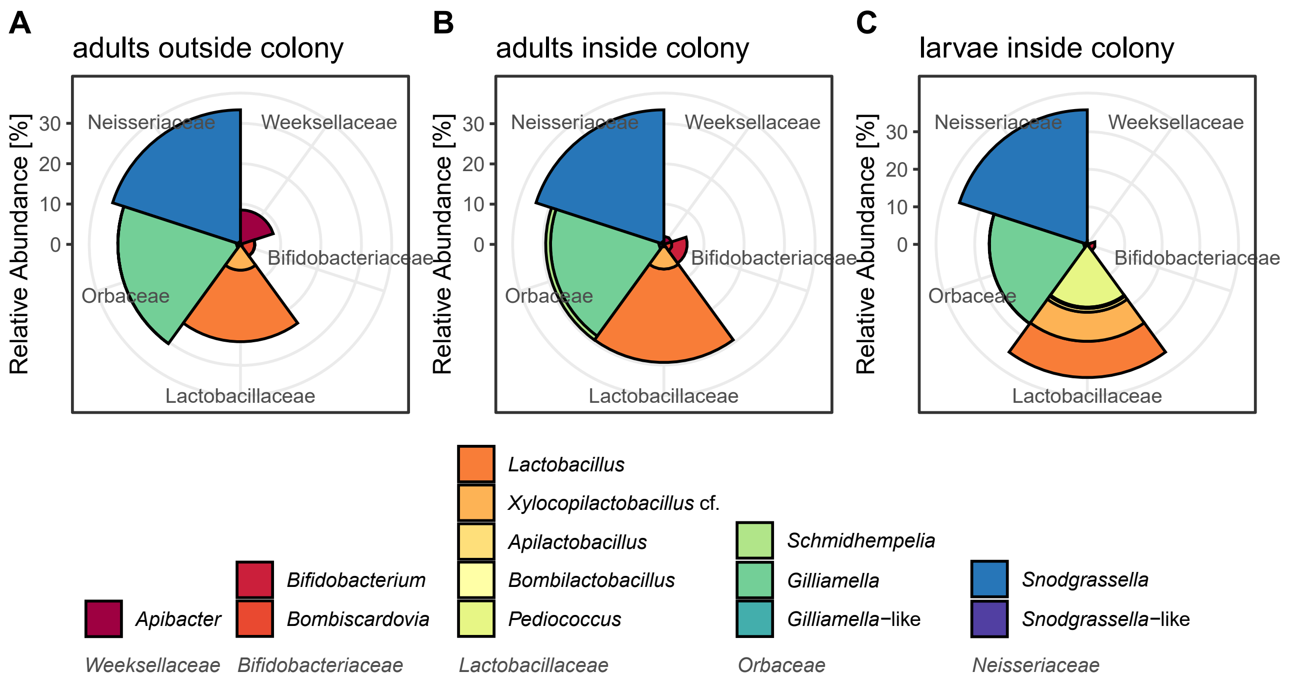

Supplemental figure S 3 Comparison of the bumble bee gut-microbiota of foraging worker with adults and larvae sampled from inside the colony.

All samples (adults outside colony, adults inside colony, larvae inside colony) were taken at the final sampling time point (‘t6’). Foraging adults (n = 10) indicate higher abundance of *Apibacter*, but lower abundance of *Bifidobacterium*. *Schmidhempelia* was only detected in adults sampled from inside the colony (n = 20). Larval samples (n = 6) show larger abundance of *Pediococcus*.

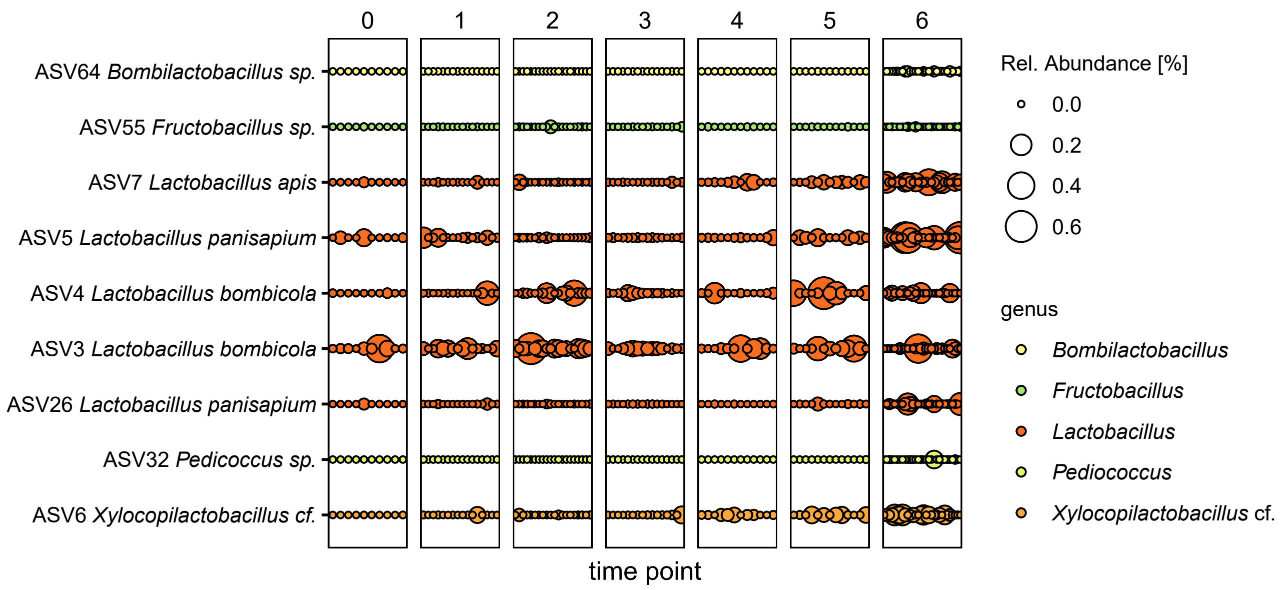

Supplemental figure S 4 ASV strain turnover within *Lactobacillaceae* over time.
Nine most abundant ASVs within the *Lactobacillaceae* are shown in their abundance dynamic within individual samples from time point 't0' to 't6'. Detailed taxonomy of ASVs obtained from Supplemental figure S 1. Increase in relative abundance over time can be primarily observed for *Lactobacillus apis* (ASV7), *L. panisapium* (ASV5, ASV26) and *Xylocopilactobacillus* cf. (ASV6). Other strains like *L. bombicola* (ASV3, ASV4) show a more erratic and variable abundance with no clear temporal trend.
